# Salvage chemotherapy after progression on immunotherapy in recurrent/metastatic squamous cell head and neck carcinoma

**DOI:** 10.3389/fonc.2024.1458479

**Published:** 2024-11-25

**Authors:** Sandra Llop, Maria Plana, Sara Tous, Angelica Ferrando-Díez, Jesús Brenes, Marc Juarez, Zara Vidales, Esther Vilajosana, Isabel Linares, Lorena Arribas, Maria Duch, Marta Fulla, Aina Brunet, Alicia Lozano, Beatriz Cirauqui, Ricard Mesía, Marc Oliva

**Affiliations:** ^1^ Department of Medical Oncology, Catalan Institute of Oncology (ICO), L’Hospitalet de Llobregat, Barcelona, Spain; ^2^ Cancer Epidemiology Research Programme, Catalan Institute of Oncology (ICO), Institut d’Investigació Biomèdica de Bellvitge (IDIBELL), L’Hospitalet de Llobregat, Barcelona, Spain; ^3^ CIBER en Epidemiología y Salud Pública (CIBERESP), Madrid, Spain; ^4^ Department of Medical Oncology, Catalan Institute of Oncology (ICO), B-ARGO group, IGTP, Badalona, Spain; ^5^ Department of Radiation Oncology, Catalan Institute of Oncology (ICO), L’Hospitalet de Llobregat, Barcelona, Spain; ^6^ Clinical Nutrition Unit, Catalan Institute of Oncology (ICO), IDIBELL, L’Hospitalet de Llobregat, University of Barcelona, Barcelona, Spain; ^7^ Department of Oral and Maxillofacial Surgery. Hospital Universitari Bellvitge, Barcelona, Spain; ^8^ Department of Otorhinolaryngology, Head and Neck Surgery, Hospital Universitari Bellvitge, Barcelona, Spain

**Keywords:** head and neck, squamous cell carcinoma, HNSCC, immunotherapy, anti-PD-(L)1, salvage chemotherapy, SCT, treatment sequencing

## Abstract

**Objectives:**

Anti-PD-(L)1 agents changed the landscape of recurrent or metastatic head and neck squamous cell carcinoma (R/M HNSCC) treatment. Previous studies showed improved response rates to salvage chemotherapy (SCT) after progression to anti-PD-(L)1 agents. This study aims to evaluate the outcomes of SCT and to identify predictors of response and survival in patients with R/M HNSCC.

**Materials and methods:**

Retrospective cohort analysis of 63 R/M patients treated with SCT after antiPD-(L1)-based therapy between January 2015 and August 2022. The overall response rate (ORR) was evaluated. Progression-free survival (PFS) and overall survival (OS) were estimated with Kaplan–Meier method. Progression-free survival 2 was calculated from anti-PD-(L)1-therapy start until progression to SCT (PFS2-I). Logistic regression and Cox regression analyses were performed to identify predictors of outcome.

**Results:**

A total of 63 patients were included: 76% were men, and median age was 60 years. PD-L1 status was available in 68% (61% positive). Up to 71% received SCT as third line or beyond. ORR to SCT was 49% with higher rates in PD-L1 positive tumors, 71% vs. 18% (p=0.001), and cetuximab-containing regimens, 68% vs. 39% (p=0.026). PD-L1 status was the only predictor of ORR in the adjusted model (OR=8.6, 95% CI 1.7–43.0). OS and PFS were 9.3 months (95% CI, 6.5–12.3) and 4.1 months (95% CI, 3.0–5.8) respectively. PFS2-I was 8.6 months (95% CI, 6.6–10.5). In the multivariate analysis, PD-L1 was the only independent factor for OS (HR=0.3; 95% CI, 0.1–0.7), PFS (HR=0.2; 95% CI, 0.1–0.5; p<0.001), and PFS2-I (HR=0.2; 95% CI 0.1–0.5; p<0.001).

**Conclusion:**

PDL1 status appeared as a strong predictor of response of efficacy for SCT after anti-PD-(L)1 agents. Patients receiving cetuximab-containing regimens trended towards greater benefit. This highlights the importance of treatment sequencing and personalized treatment strategies.

## Introduction

The prognosis of patients with recurrent or metastatic head and neck squamous cell carcinoma (R/M HNSCC) remains poor, with a guarded median overall survival (OS) ranging from 10 months to 14 months (1). The combination of cetuximab, 5-fluorouracil, and platinum has been the standard of care in the first-line setting over the past decade (2). Upon progression, the only treatment option was single-agent chemotherapy, with limited response rates and a median survival of 6 months (3, 4).

Anti-PD-1 agents have emerged as the new standard of care in R/M HNSCC, as they showed an OS improvement in both first-line and platinum-refractory settings (5). The Checkmate 141 and Keynote 040 trials demonstrated increased OS in platinum-refractory R/M HNSCC, using nivolumab and pembrolizumab, respectively, although in the Keynote 040 the benefit was limited to patients with PD-L1-positive disease (6, 7). In the first-line setting, pembrolizumab alone or in combination with platinum-based chemotherapy improved OS compared to the EXTREME regimen in patients with PD-L1-positive disease (1). However, none of these trials demonstrated benefit in terms of PFS. Additionally, ORR to anti-PD-1 agents alone was modest. The OS improvement therefore may be partly explained by subsequent treatments following anti-PD-1, possibly due to enhanced sensitivity to salvage chemotherapy (SCT).

In this regard, the effect of first line on subsequent anticancer therapy was explored in the Keynote 048 study: longer PFS2-I [defined as time from anti-PD-(L)1-based therapy start until progression to SCT or death from any cause] was observed in the PD-L1 CPS ≥20 and CPS ≥1 patients treated with pembrolizumab alone, and in total population, regardless of PD-L1 expression, treated with the combination of chemotherapy and pembrolizumab when compared to cetuximab plus chemotherapy (8).

In other tumor types, such as non-small-cell lung cancer (NSCLC) and urothelial cancer, several trials have demonstrated enhanced responses to SCT following progression on immune checkpoint inhibitors (ICI), surpassing historical benchmarks (9, 10). In R/M HNSCC, a few retrospective series with limited number of patients have shown increased response rates to chemotherapy after anti-PD-1 treatment, compared to historical data (**Table 1**) (11–20).

**Table 1:** Summary of retrospective studies evaluating the role of salvage chemotherapy after anti-PD-1 treatment in R/M head and neck cancer.

Study reference	N	ORR	PFS	OS
Koyama T. et al., 2024 (11)	35	69.6%	5.5 months	13.3 months
Tanaka H. et al., 2023 (12)	59	62.7%	4.6 months	17.1 months
Wakasaki T. et al., 2022 (13)	52	53%	7.4 months	11.9 months
Cabezas S. et al., 2021 (14)	23	56.5%	6 months	12 months
Kurosaki T. et al., 2021 (15)	22	40.9%	5.2 months	14.5 months
Suzuki S. et al., 2020 (16)	18	44.4%	3.8 months	9.6 months
Kacew A. et al., 2020 (17)	60	27%	3.3 months	9.8 months
Pestana R. et al., 2020 (18)	43	42%	4.2 months	8.4 months
Ueki Y. et al., 2020 (19)	21	52.4%	5.4 months	12.9 months
Saleh K. et al., 2019 (20)	82	53%	3.6 months	7.8 months

N, number of patients included; ORR, overall response rate; PFS, progression-free survival; OS, overall survival; m, months

Understanding the response patterns and potential predictors of favorable SCT outcomes could have significant implications in tailoring the best treatment sequence for patients with R/M HNSCC. Moreover, it is unclear whether the addition of Cetuximab to SCT leads to improved responses and survival in this setting.

The aim of this study was to evaluate the efficacy of SCT with/without Cetuximab in patients with R/M HNSCC after progression on anti-PD-(L)1 alone or in combination with other immunotherapies and to identify predictors of treatment outcome in terms of ORR, PFS, PFS2-I, and OS.

## Materials and methods

### Study population and design

Retrospective multicenter cohort analysis of R/M HNSCC patients treated with SCT following disease progression on or after anti-PD-(L)1 agents was conducted between January 2015 and August 2022 in two monographic cancer centers [Institut Català d’Oncologia (ICO) Badalona and ICO-Hospitalet]. Selection criteria for inclusion were as follows: 1) recurrent/metastatic HNSCC, 2) progression on or after treatment with anti-PD-(L)1 agents used at any line, and 3) received SCT after anti-PD-(L)1 agents. Patient demographics, disease characteristics including PD-L1 status, treatment characteristics, and response were retrospectively reviewed by two independent investigators. A third rater reviewed 10% of the data to ensure accuracy.

PD-L1 expression was measured using an immunohistochemistry assay (PD-L1 IHC 22C3 pharmDx; Dako North America, Carpinteria, CA). PD-L1 was considered positive when ≥1% of cells showed partial membrane staining, according to the Combined Positive Score (CPS) or the Tumor Proportion Score (TPS) (21, 22).

Human papillomavirus (HPV) status was evaluated in oropharyngeal cancer, and tumors were considered HPV-related if HPV-DNA polymerase chain reaction and p16^INK4a^ immunohistochemistry determination were both positive.

Platinum-refractory disease was defined as progression on platinum-based chemotherapy for advanced disease or relapsing within 6 months of platinum-based therapy with curative intent. Patients with primary resistance to immunotherapy (refractory disease) were defined as those progressing within 3 months since the start of immunotherapy for recurrent/metastatic disease, in concordance with SITC guidelines (23).

Tumor response to SCT was assessed according to the Response Evaluation Criteria in Solid Tumors (RECIST), version 1.1. The objective response rate (ORR) was defined as the proportion of patients who exhibited complete response (CR) or partial response (PR) as the best response. The disease control rate (DCR) was defined as the proportion of patients who exhibited CR, PR, or stable disease (SD) as the best response. Response evaluation was performed using computed tomography every 8–12 weeks as per institutional protocols.

### Statistical analysis

Logistic regression analysis was performed to identify determinants for ORR. Crude and adjusted odds ratio (OR) and 95% confidence intervals (CIs) were calculated, and models were compared using log-likelihood ratio test.

PFS was defined as the time from the initiation of SCT until disease progression, death due to any cause, or the cutoff date. OS was defined as the time from the initiation of SCT until death due to any cause or the cutoff date. PFS2-I was defined as the time from the initiation of anti-PD-(L)1 agents until disease progression on subsequent SCT, death due to any cause, or the cutoff date. Adverse events (AEs) were recorded using the National Cancer Institute Common Terminology Criteria for Adverse Events, version 5.0.

Overall survival, PFS, and PFS2-I were estimated using the Kaplan–Meier method and log-rank test for comparisons between curves. Patients who were lost to follow-up or were still alive without progression by the end of the study were censored at the date of last follow-up. A multivariate Cox regression model was performed to identify predictors of efficacy to SCT in terms of PFS, PFS2-I, and OS. Crude and adjusted hazard ratios (HR) and its 95% CI were calculated. Models were compared using log-likelihood ratio test, and proportional hazards assumption was assessed both by the proportional hazard test and the scaled Shoenfeld residuals.

Variables considered as potential determinants of efficacy in terms of ORR, OS, PFS, and PFS2-I were age, PD-L1 status, baseline ECOG (at SCT initiation), number of previous lines, platinum resistance, and cetuximab-containing regimen, and were included in the univariate analysis. Those variables that showed a statistically significant impact in terms of efficacy were included in the multivariate model.

All analyses were performed using STATA (StataCorp, 2020, Stata Statistical Software: Release 16.1, College Station, TX: StataCorp LLC), and the statistical significance threshold was set at 0.05.

## Results

### Cohort characteristics

From January 2015 to August 2022, a total of 63 patients who received SCT met eligibility criteria and were included in the analysis. Baseline cohort characteristics are summarized in **Table 2**. Most patients were men (76%) and former/current smokers (86%). The most prevalent primary tumor location was the oral cavity (33%), followed by larynx (27%) and oropharynx (25%). Only 37.5% (N=6) of oropharyngeal cases were HPV-related. PD-L1 status was available in 68.3% cases, of which 26 (60.5%) were positive (≥1) either by CPS or TPS. A total of 10 (15.9%) patients presented with platinum-refractory disease. Two-thirds of patients had received two lines of therapy for R/M disease prior to SCT. Half of the patients received a PD-(L)1 inhibitor as monotherapy (49%), while the other half received a combination of PD-(L)1 inhibitor plus another immuno-oncology agent. The median PFS and OS to antiPD-(L)1 based therapy were 2.6 months (95% CI, 2.2–2.9) and 12.9 months (95% CI, 9.7–21.3), respectively. The ORR was 12.7%, and the DCR was 38.1%. No significant differences in ORR were observed by PD-L1 status (positive vs. negative): 17.7% vs. 7.7%, respectively (p = 0.369). There were 41 patients (65.1%) with immunotherapy-refractory disease.

**Table 2:** Patient characteristics at SCT initiation.

	N (%)
Age: median (range)	60.3 (24.7–78.6)
Sex: Male vs. female	48 (76.2) vs. 15 (23.8)
ECOG: 1 2	39 (81.2) 9 (18.8)
Tumor location: Oral cavity Larynx Oropharynx; HPV-related Hypopharynx Nasal cavity Cervical squamous cell carcinoma with unknown origin	21 (33.3) 17 (27.0) 16 (25.4); 6 (37.5) 7 (11.1) 1 (1.6) 1 (1.6)
Status PD-L1: Positive Negative	26 (60.5) 17 (39.5)
Platinum-refractory status: Platinum-refractory disease (≤6 months) Platinum-sensitive disease	10 (15.9) 53 (84.1)
Immunotherapy-refractory status Immunotherapy-refractory disease (≤ 3months) No immunotherapy-refractory disease	41 (65.1) 22 (34.9)
Number of line for SCT: 2 3	18 (28.6) 39 (61.9)
>=4	6 (9.5)
Anti-PD-(L)1 containing treatment received: PD-(L)1 inhibitor alone PD-(L)1 inhibitor + immunomodulator PD-(L)1 inhibitor + another checkpoint inhibitor	31 (49.2) 22 (34.9) 10 (15.9)
SCT regimen:	
Cetuximab-containing regimens:	
- EXTREME	12 (19.1)
- Paclitaxel + cetuximab	11 (17.5)
- Carboplatin + cetuximab	1 (1.6)
Non-cetuximab regimens:	
- Paclitaxel	34 (54.0)
- Gemcitabine	2 (3.2)
- Methotrexate	1 (1.6)
Antibody drug conjugate (clinical trial)	2 (3.2)

SCT, salvage chemotherapy; HPV, human papilloma virus

### Efficacy of salvage chemotherapy following antiPD-(L)1 treatment

Chemotherapy regimens used as SCT are described in **Table 2**. A total of 34 patients (54%) received paclitaxel monotherapy. Regimens including chemotherapy plus cetuximab were administered in 33 patients (36.6%).

Radiologic response evaluation was available in 61 patients (not available in two cases because of clinical deterioration before radiological evaluation of response related to infectious diseases). Of those, ORR to SCT was 49.2% with nine (14.8%) complete responses. ORR was significantly higher in the PD-L1-positive compared to PD-L1-negative tumors: 70.8% vs. 17.7% (p= 0.001), and for regimens containing cetuximab vs. no cetuximab: 68.2% vs. 38.5% (p= 0.026). DCR was 55.6% for the total cohort and was greater in the PD-L1-positive compared to PD-L1-negative tumors: 85.0% vs. 66.7% (p = 0.002).

Median duration of follow-up from initiation of SCT to data cutoff or death, whichever occurred first, was 7.6 months (0.3–71.1). PFS and OS were 4.1 months (95% CI, 3.0–5.8) and 9.3 months (95% CI, 6.5–12.3 months), respectively. PFS 2 was 8.6 months (95% CI, 6.6–10.5 months).

**Figure 1:**
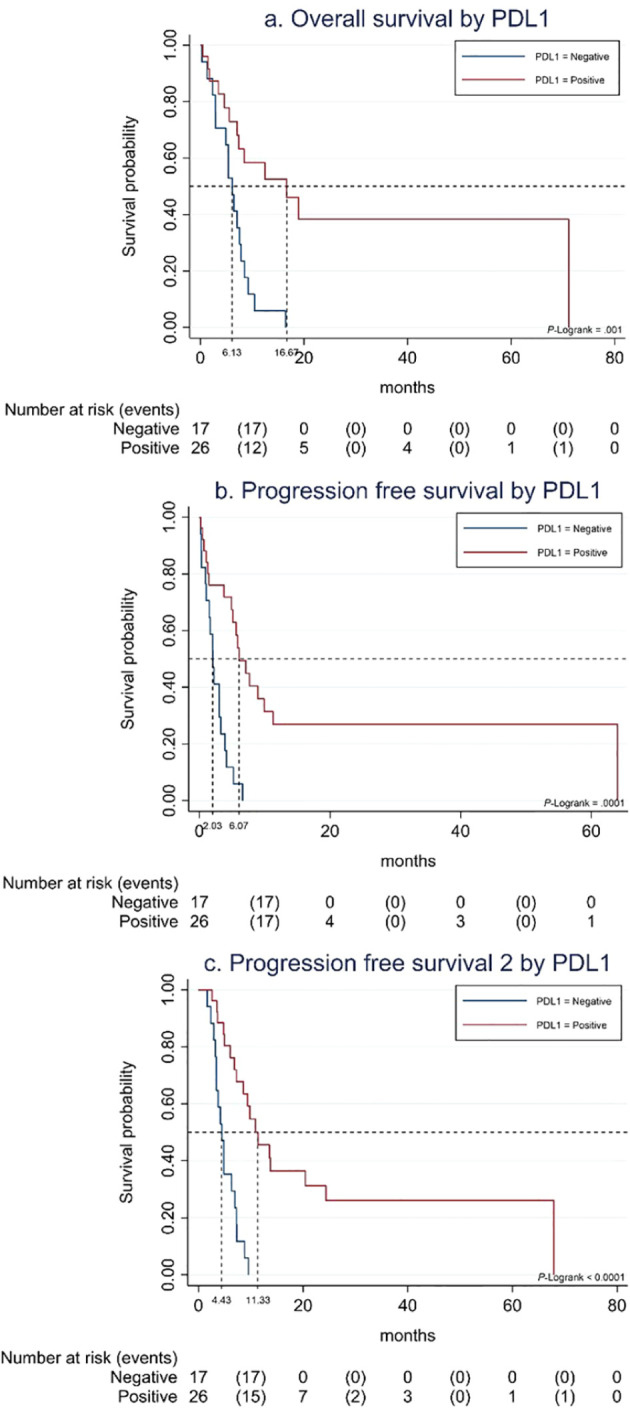
**(A)** Overall survival to SCT by PDL-1 status. **(B)** Progression-free survival to SCT by PDL-1 status. **(C)** Progression-free survival 2 to SCT by PDL-1 status.

PD-L1-positive status was associated with better outcome to SCT in terms of PFS [6.1 months (95% CI, 4.9–9.9) vs. 2.0 months (95% CI, 1.0–3.3); p< 0.001], OS [16.7 months (95% CI, 5.6–NR) vs. 6.1 months (95% CI, 2.9–7.9); p 0.001], and PFS2-I [11.3 months (95% CI, 7.2–24.3) vs. 4.4 months (95% CI, 3.3–6.9); p< 0.001] (**Figure 1**). OS was significantly higher in patients with Eastern Cooperative Oncology Group (ECOG) performance status 1 vs. 2 [12.3 months (95% CI, 7.1–19.0) vs. 5.4 months (95% CI, 0.3–8.5); p 0.001], with no differences in PFS nor PFS2-I.

There were no significant differences in SCT outcomes between platinum sensitive vs. refractory patients in terms of ORR [51.0% vs. 40.0%; p=0.525], PFS [4.13 months (95% CI, 3–5.8) vs. 2.4 months (95% CI, 0.7–NR); p=0.620], and OS [8.5 months (95% CI, 6.5–12.2) vs. 10.5 months (95% CI, 1.8–NR); p=0.685].

Patients receiving regimens containing cetuximab trended towards greater PFS [6.1 months (95% CI, 1.5–9.9) vs. 3.8 months (95% CI, 2.0–5.2); p=0.078] and PFS2-I [10.9 months (95% CI, 7.2–20.4) vs. 6.9 months (95% CI, 4.9–9.4), p=0.063] but not OS [12.5 months (95% CI, 5.6–NR) vs. 8.4 months (95% CI, 6.1–10.5); p=0.255] when compared to non-cetuximab regimens (**Supplementary Figure S1**). Taxanes-containing regimens did not show greater PFS, OS, or PFS2-I compared to those without taxanes (p=0.478, p=0.197, and p=0.548 respectively). A subgroup analysis was performed in patients who received Paclitaxel + Cetuximab vs. weekly Paclitaxel monotherapy as SCT regimen. Patients receiving Paclitaxel + Cetuximab showed increased ORR (72.7% vs. 36.4%, p = 0.036), PFS [9.9 months (95% CI, 0.7–NR) vs. 3.8 months (95% CI, 2–5.1), p=0.0340], and OS [12.5 months (95% CI, 1.8–NR) vs. 7.6 months (95% CI, 5.4–10.4), p=0.119] compared to those receiving Paclitaxel monotherapy.

In the subgroup of patients diagnosed with oropharyngeal cancer, HPV status positive vs. negative did not have an impact in SCT efficacy: ORR, 50.0% vs. 62.5% (p=0.960); PFS, 2.2 months (95% CI, 0.17–NR) vs. 5.1 (95% CI, 1.0–9.1) (p=0.698); and OS, 10.4 months (95% CI, 1.8–NR) vs. 12.0 months (95% CI, 1.3–NR) (p 0.999).

No differences in SCT outcomes were observed according to response to prior immunotherapy (responders vs. refractory) between immunotherapy responders vs. non-responders in terms of ORR (50.0% vs. 47.3%; p=0.859), PFS [3.9 months (95% CI, 0.17–8.9) vs. 4.9 months (95% CI, 2.2–5.8); p=0.892], and OS [12.2 months (95% CI, 6.4–NR) vs. 8.4 months (95% CI, 5.6–12.0); p=0.653]. A subgroup analysis was performed in patients with immunotherapy-refractory disease evaluable for response (N=40): ORR was 40.0%, with eight patients (20%) achieving CR. PFS was 3.8 months (95% CI, 1.8–5.1), OS was 7.4 months (95% CI, 5.4–9.3), and PFS2-I was 6.3 months (95% CI, 4.2–7.2).

### Predictors of response and survival to SCT

Only PD-L1 status showed a significant association with improved ORR in the logistic regression analysis. Patients with PD-L1-positive disease exhibited higher ORR to SCT compared to PD-L1-negative patients (OR=8.61; 95% CI, 1.72–43.00), after adjusting for PD-L1 and cetuximab-containing regimen (**Table 3**).

**Table 3:** Determinants of ORR.

Characteristic	ORR		Crude OR			Adjusted		
	Yes	(%)	OR	(95%CI)	p-value	OR	(95% CI)	p-value
**Age**					0.3441			
Mean (SD)	59.30	(10.27)	0.97	(0.92–1.03)				
**PDL1**					**0.0025**			**0.0187**
Negative	3	(17.65)	Ref.			Ref.		
Positive	17	(70.83)	11.33	(2.46–52.15)		8.61	(1.72–43.0)	
Unknown	10	(50.00)	4.67	(1.02–21.43)		3.80	(0.78–18.44)	
**ECOG baseline**					0.1627			
1	22	(57.99)	4.12	(0.73–23.15)				
2	2	(25.00)	Ref.					
Unknown	6	(40.00)	2.00	(0.30–13.44)				
**Number of lines**					0.1058			
2	11	(67.71)	2.04	(0.63–6.64)				
3	18	(47.37)	Ref.					
>=4	1	(16.67)	0.22	(0.02–2.09)				
**Cetuximab-containing regimen**					**0.0245**			0.3146
No	15	(38.46)	Ref.			Ref.		
Yes	15	(68.18)	3.43	(1.14–10.35)		1.87	(0.55–6.36)	

ORR, overall response rate; SD, standard deviation; OR, odds ratio; CI, confidence interval; PDL1, Programmed Death-Ligand 1; ECOG, Eastern Cooperative Oncology Group.

Mean age for non-responders is 61.47 (SD=7.81) non-significantly different from responders (ANOVA p-value = 0.3555). p-value corresponds to the log-likelihood test for comparisons between models with and without the characteristic.

Bold values indicate statistically significant values.

In the multivariate Cox regression analysis for OS (**Supplementary Table S1**), only PD-L1 status was an independent prognostic factor for OS (HR=0.30; 95% CI, 0.13–0.70; p=0.006) after adjusting for ECOG. PD-L1 status also significantly impacted on PFS (HR=0.23; 95% CI, 0.11–0.48; p=0.001). In terms of PFS2-I, only PDL1 status was a significant predictor for improved PFS2-I (HR = 0.20; 95% CI, 0.08–0.46; p=0.001), after adjusting for ECOG and the cetuximab-containing regimen.

In the subgroup analysis for patients receiving Paclitaxel + Cetuximab vs. weekly Paclitaxel monotherapy, PD-L1status remained the only prognostic factor for OS (HR, 0.33; 95% CI, 0.12–0.90; p = 0.030), PFS (HR, 0.25; 95% CI, 0.09–0.70; p = 0.008), and PFS2-I (HR, 0.21; 95% CI, 0.07–0.58; p = 0.003), after adjusting for ECOG and cetuximab-containing regimen.

## Discussion

In this retrospective analysis, we evaluated the efficacy of SCT plus/minus cetuximab following progression on or after anti-PD-(L)1 agents in patients with R/M HNSCC. The observed ORR was close to 50% and was higher in patients with PD-L1-positive disease and when using cetuximab-based regimens. In this pre-treated patient population, PFS and OS were 4.1 months (95% CI, 3.0–5.8) and 9.3 months (95% CI, 6.5–12.3), respectively. PFS2-I was 8.6 months (95% CI, 6.6–10.5). SCT efficacy remained notable in immunotherapy-refractory patients, with an ORR of 40% and up to eight patients (20%) achieving a CR. These results are of relevance when compared to historical data in the pre-immunotherapy era, particularly when more than two-thirds received SCT as a third line of treatment or beyond.

These data are in line with other retrospective series evaluating the role of SCT after anti-PD-(L)1 treatment (see **Table 1**). Notably, in a French retrospective cohort of 82 patients, Saleh et al. reported an ORR of 30% to SCT, which increased to 53% in cetuximab-based chemotherapy regimens. The PFS was 3.6 months in this cohort (20). Similarly, in a retrospective study conducted by Pestana et al., involving 43 patients with R/M HNSCC who received SCT after anti-PD-1 treatment, an ORR of 42% and a PFS of 4.2 months were observed. Interestingly, the ORR with single agent cetuximab was 37.5% without significant differences compared to chemo-containing regimens (18). In contrast, other series such as Koyama et al., Tanaka et al., and Cabezas et al. reported higher ORR, PFS, and OS measures. These differences could potentially be attributed to factors such as patients being less heavily pretreated (with a high percentage receiving immune checkpoint inhibitors in the first-line setting) and the consistent use of combined chemotherapy with cetuximab in all patients.

The role of PD-L1 as an independent prognostic factor and predictor of benefit to antiPD(L)-1 agents in R/M HNSCC is well-established. However, it is less clear whether it has role in predicting response to SCT. In our cohort, positive PD-L1 expression significantly correlated with improved ORR to SCT, which is in concordance with the results reported by Ueki et al. (19). Nonetheless, controversies exist in the literature regarding the prognostic significance of PD-L1 in this context, probably influenced by small sample sizes and heterogeneous populations in the published series. Immunotherapy-based treatments can induce immune-mediated changes in the tumor microenvironment, especially in PD-L1 positive tumors, creating an inflamed phenotype characterized by increased immune cell infiltration and subsequently enhanced PD-L1 expression. A hot tumor microenvironment has been associated with improved responses to chemotherapy and increased susceptibility to immune cell-mediated cytotoxicity (24). As a result, the combination of ICI-induced microenvironment changes and the subsequent cytotoxic chemotherapy effect might act synergistically and improve therapeutic outcomes with a greater effect in PD-L1-positive patients (25). Moreover, the phenomenon of induced immunogenic cell death might contribute to increased sensitivity to chemotherapy. Immunogenic cell death is known to release damage-associated molecular patterns and tumor-associated antigens (TAAs) from dying tumor cells, triggering an immune response against the tumor, enhancing antigen presentation and priming of cytotoxic T-cells, ultimately leading to immune-mediated tumor cells death. Therefore, when chemotherapy is subsequently administered, the activated T cells may respond to released tumor-associated antigens and kill tumor cells effectively (26).

On the other hand, chemotherapy agents have been shown to deplete regulatory T cells (T regs) and myeloid-derived suppressor cells (MDSCs), which contribute to immune suppression and promote tumor growth. By reducing these suppressive cell populations, chemotherapy might reduce immunosuppression in the tumor microenvironment allowing a more favorable immune landscape. Consequently, the activated cytotoxic T cells, unleashed by immunotherapy and primed to recognize TAAs, may encounter reduced immune inhibition, further promoting tumor cell killing (27).

We found that patients receiving cetuximab plus SCT showed greater ORR and a trend to better PFS and OS when compared to chemotherapy agents alone, as had been previously suggested by other small retrospective series (14).

Besides its direct EGFR inhibition, cetuximab has been shown to induce an antibody-dependent cellular cytotoxicity (ADCC) via natural killer (NK) activation (28, 29). Administration of cetuximab in combination with ICI enhances cetuximab-mediated ADCC *in vitro* (30) and the combination of pembrolizumab with cetuximab in a phase II trial involving R/M HNSCC patients demonstrated increased ORR when compared to historical responses of both agents when given as monotherapy, indicating an additive or potentially synergistic effect of both drugs combined (31). In this regard, several trials are currently investigating cetuximab in combination with different IO agents. The increased efficacy of SCT with cetuximab following antiPD-1 therapy could be due to a delayed overlapping effect of this treatment sequences.

In the multivariate analysis evaluation, no independent impact on ORR, OS, PFS, or PFS2-I was observed for age, ECOG, or number of previous lines. Although patients with ECOG 1 exhibited a trend towards improved OS, this trend did not reach statistical significance when adjusted for PD-L1 status. No differences in outcomes were found by chemotherapy regimens (taxanes vs. other including platinum) probably due to the small sample size and the fact that non-taxane regimens were more frequently used in earlier lines of treatment.

The authors acknowledge the inherent limitations of a retrospective study and the small sample size, which includes heavily pretreated patients. Furthermore, the heterogeneity of the SCT regimens used in the studied population might have impacted our results. Another limitation stems from the unknown PD-L1 status in up to a third of patients, and the heterogeneity in the evaluation of PD-L1 status by either TPS or CPS. Of note, CPS was not the standard at the time these patients were treated. The modest cohort size may have limited the ability to detect differences between platinum-sensitive and platinum-refractory patients. Furthermore, most patients receiving non-cetuximab regimens were treated with chemotherapy monotherapy, potentially affecting the higher perceived efficacy of cetuximab-containing regimens that incorporated chemotherapy combinations. Given the retrospective nature of this study, patient-centered efficacy measures, such as quality of life and patient-reported outcomes, are not available. These limitations underscore the need for prospective clinical trials with larger sample sizes and control arms. Moreover, future investigations should explore potential biomarkers beyond PD-L1 to identify patients who would most benefit from this sequential treatment strategy.

In conclusion, this study confirms the increased efficacy of SCT after anti-PD-(L)1 agents in patients with pre-treated R/M HNSCC, while it identifies potential predictors of benefit that may aid treatment decision-making in clinical practice. The observed increased ORR compared to pivotal trials, particularly in PD-L1-positive patients and cetuximab-based regimens, indicate a promising direction for personalized treatment sequence strategies. Larger and prospective studies are needed to further understand the underlying mechanisms driving these responses and guide therapeutic approaches.

## Data Availability

The original contributions presented in the study are included in the article/**Supplementary Material**. Further inquiries can be directed to the corresponding author.
