# Supplementary material for: Salvage chemotherapy after progression on immunotherapy in recurrent/metastatic squamous cell head and neck carcinoma
Source: Front Oncol. 2024 Nov 25;14:1458479. doi: 10.3389/fonc.2024.1458479 (PMC11625818; doi:10.3389/fonc.2024.1458479)
Supplement: Supplementary file 1 [file DataSheet1.docx]

Supplementary Material

Salvage chemotherapy after progression on immunotherapy in recurrent/metastatic squamous cell head and neck carcinoma.

Sandra Llop^1^, Maria Plana^1^, Sara Tous^2, 3^, Angelica Ferrando-Díez^4^, Jesús Brenes^1^, Marc Juarez^5^, Zara Vidales^1^, Esther Vilajosana^1^, Isabel Linares^5^, Lorena Arribas^6^, Maria Duch^7^, Marta Fulla^8^, Aina Brunet^8^, Alicia Lozano^5^, Beatriz Cirauqui^4^, Ricard Mesía^4^, Marc Oliva^1^

*** Correspondence:** Corresponding Author: [moliva@iconcologia.net](https://correu.iconcologia.net/owa/redir.aspx?C=q4Y-HCs7jUmYqIkb16-N20W3iYrcoNcI0kpM1XFcYZ6YYVWzhwbGK4ZdPRTIfANNxoxxcWc2uag.&URL=mailto%3amtaberna%40iconcologia.net)

# Supplementary Figures and Tables

**Supplementary material**


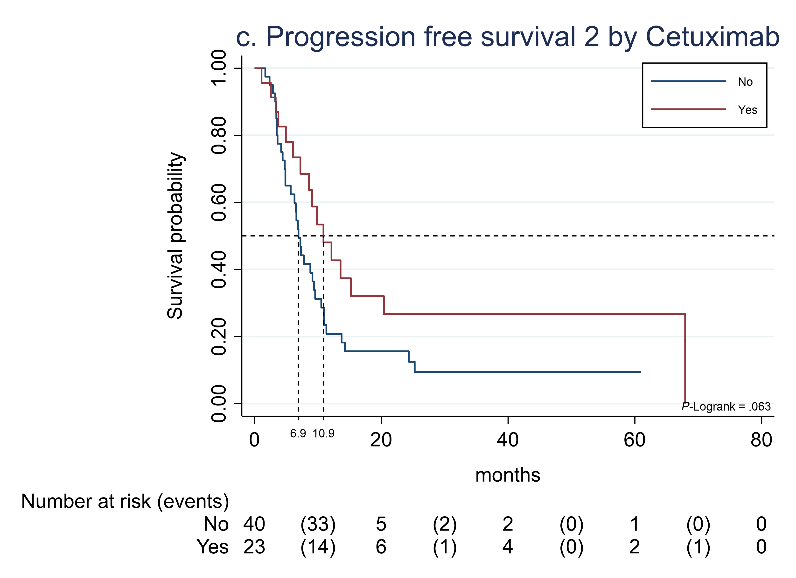

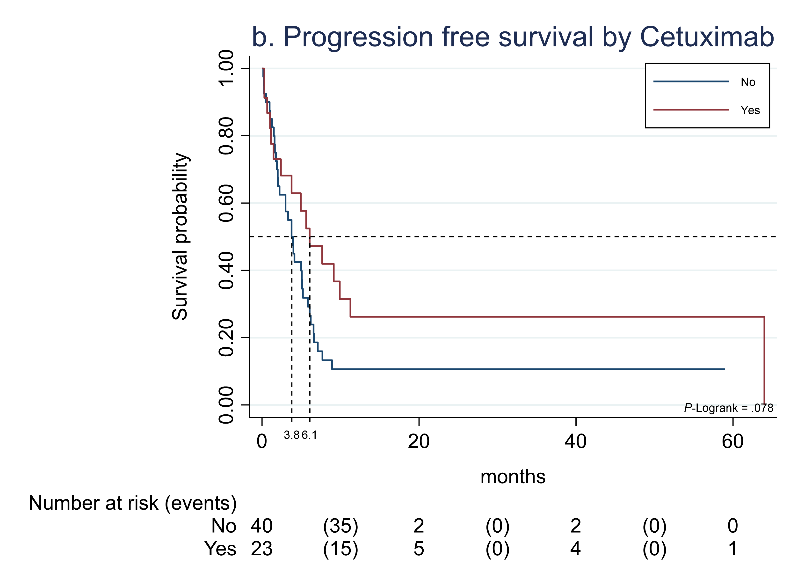

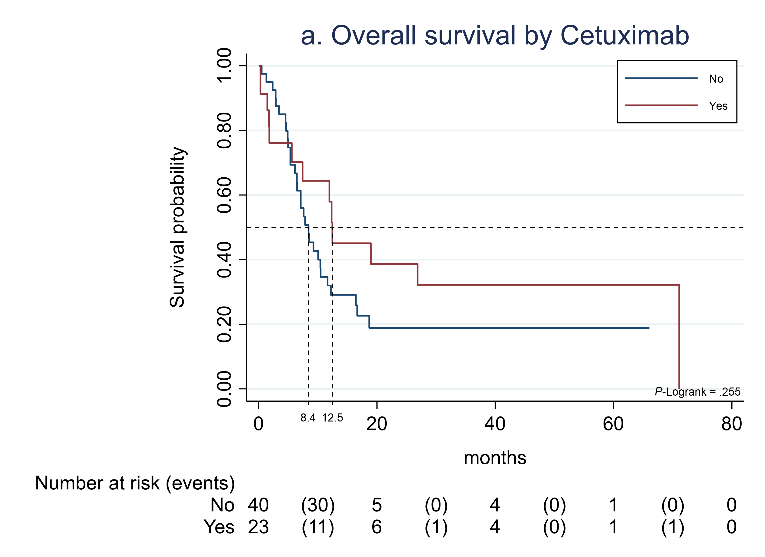


Supplementary Figure S1. 2A) Overall survival to SCT by Cetuximab-containing regimen. 2B) Progression free survival to SCT by Cetuximab-containing regimen. 2C) Progression free survival 2 to SCT by Cetuximab-containing regimen.

Supplementary Table S1. Univariate and Multivariate analysis for Overall survival, Progression free survival and Progression free survival 2.

|  | **OS** | | | | | | | **PFS** | | | | **PFS2-I** | | | | | | |
| --- | --- | --- | --- | --- | --- | --- | --- | --- | --- | --- | --- | --- | --- | --- | --- | --- | --- | --- |
|  |  | Crude | | | Adjusted | | |  | Crude | | |  | Crude | | | Adjusted | | |
| Characteristics | Deaths,  n/patients,  N | HR | (95% CI) | p-value | HR | (95% CI) | p-value | Event,  n/patients,  N | HR | (95% CI) | p-value | Event,  n/patients,  N | HR | (95% CI) | p-value | HR | (95% CI) | p-value |
| All patients | 43/63 |  |  |  |  |  |  | 51/63 |  |  |  | 51/63 |  |  |  |  |  |  |
| **Age** |  | 1.004 | (0.97-1.04) | 0.8057 |  |  |  |  | 0.99 | (0.96-1.02) | 0.5398 |  | 0.99 | (0.96-1.03) | 0.6401 |  |  |  |
| **PDL1** |  |  |  | **0.0010** |  |  | **0.0056** |  |  |  | **0.0006** |  |  |  | **<0.0001** |  |  | **0.0007** |
| Negative | 17/17 | Ref. |  |  | Ref. |  |  | 17/17 | Ref. |  |  | 17/17 | Ref. |  |  | Ref. |  |  |
| Positive | 13/26 | 0.25 | (0.11-0.54) |  | 0.30 | (0.13-0.70) |  | 18/26 | 0.23 | (0.11-0.48) |  | 18/26 | 0.17 | (0.08-0.37) |  | 0.20 | (0.08-0.46) |  |
| Unknown | 13/20 | 0.28 | (0.13-0.62) |  | 0.33 | (0.14-0.76) |  | 16/20 | 0.33 | (0.16-0.69) |  | 16/20 | 0.22 | (0.10-0.47) |  | 0.24 | (0.10-0.56) |  |
| **ECOG baseline** |  |  |  | **0.0208** |  |  | 0.3000 |  |  |  | 0.1357 |  |  |  | 0.0714 |  |  | 0.3383 |
| 1 | 24/39 | 0.26 | (0.11-0.62) |  | 0.49 | (0.19-1.22) |  | 30/39 | 0.45 | (0.20-1.00) |  | 30/39 | 0.41 | (0.18-0.92) |  | 0.78 | (0.33-1.87) |  |
| 2 | 8/9 | Ref. |  |  | Ref. |  |  | 8/9 | Ref. |  |  | 8/9 | Ref. |  |  | Ref. |  |  |
| Unknown | 11/15 | 0.33 | (0.12-0.85) |  | 0.63 | (0.23-1.79) |  | 13/15 | 0.67 | (0.27-1.64) |  | 13/15 | 0.68 | (0.27-1.67) |  | 1.29 | (0.49-3.38) |  |
| **Number of lines** |  |  |  | 0.6743 |  |  |  |  |  |  | 0.5497 |  |  |  | 0.3009 |  |  |  |
| 2 | 11/18 | 0.99 | (0.48-2.05) |  |  |  |  | 13/18 | 0.76 | (0.39-1.47) |  | 13/18 | 0.72 | (0.37-1.39) |  |  |  |  |
| 3 | 27/39 | Ref. |  |  |  |  |  | 32/39 | Ref. |  |  | 32/39 | Ref. |  |  |  |  |  |
| >=4 | 5/6 | 1.56 | (0.60-4.08) |  |  |  |  | 6/6 | 1.27 | (0.53-3.07) |  | 6/6 | 1.58 | (065-3.83) |  |  |  |  |
| **Cetuximab-containing regimen** |  |  |  | 0.2469 |  |  |  |  |  |  | 0.0738 |  |  |  |  |  |  | 0.5931 |
| No | 30/40 | Ref. |  |  |  |  |  | 35/40 | Ref. |  |  | 35/40 | Ref. |  | 0.0583 | Ref. |  |  |
| Yes | 13/23 | 0.68 | (0.34-1.33) |  |  |  |  | 16/23 | 0.58 | (0.31-1.07) |  | 16/23 | 0.57 | (0.31-1.04) |  | 0.83 | (0.43-1.63) |  |

Abbreviation: HR=hazard ratio. Log-likelihood ratio test. CI: Confidence interval. Event was defined as progression or death, whatever occurs first.

**
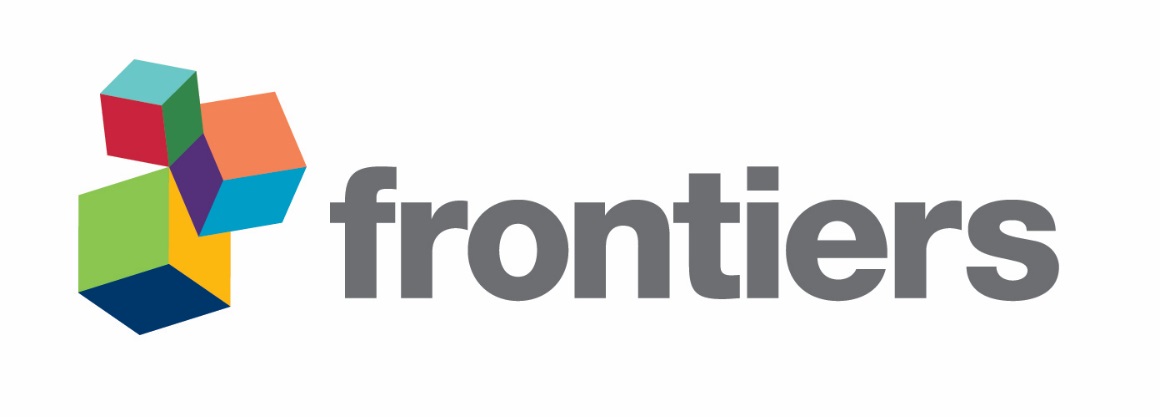
**
